# Nicotinamide N-methyltransferase (NNMT) regulates the glucocorticoid signaling pathway during the early phase of adipogenesis

**DOI:** 10.1038/s41598-023-34916-z

**Published:** 2023-05-22

**Authors:** Annalisa Roberti, Juan Ramon Tejedor, Irene Díaz-Moreno, Virginia López, Pablo Santamarina-Ojeda, Raúl F. Pérez, Rocío G. Urdinguio, Carmen Concellón, María Luz Martínez-Chantar, Juan Luis Fernández-Morera, Antonio Díaz-Quintana, Vicente del Amo, Agustín F. Fernández, Mario F. Fraga

**Affiliations:** 1 Nanomaterials and Nanotechnology Research Center (CINN), Spanish National Research Council (CSIC), 33940 El Entrego, Spain; 2 Foundation for Biomedical Research and Innovation in Asturias (FINBA), 33011 Oviedo, Spain; 3 Health Research Institute of Asturias (ISPA), Av. del Hospital Universitario, 33011 Oviedo, Asturias Spain; 4 University Institute of Oncology (IUOPA), University of Oviedo, 33006 Oviedo, Spain; 5 Center for Biomedical Network Research on Rare Diseases (CIBERER), 28029 Madrid, Spain; 6 Institute for Chemical Research (IIQ), Scientific Research Centre Isla de la Cartuja (cicCartuja), University of Seville – Spanish National Research Council (CSIC), Seville, Spain; 7 Department of Organic and Inorganic Chemistry, University of Oviedo, Oviedo, Spain; 8 Liver Disease Lab, Center for Cooperative Research in Biosciences (CIC bioGUNE), Basque Research and Technology Alliance, Derio, Spain; 9 Centro de Investigación Biomédica en Red de Enfermedades Hepáticas y Digestivas (CIBERehd), Carlos III National Health Institute, Madrid, Spain; 10 Endocrinology and Nutrition Department, Hospital Vital Alvarez Buylla (HVAB), 33611 Mieres, Spain

**Keywords:** Cell biology, Chemical biology, Molecular biology

## Abstract

Obesity is associated with adipose tissue dysfunction through the differentiation and expansion of pre-adipocytes to adipocytes (hyperplasia) and/or increases in size of pre-existing adipocytes (hypertrophy). A cascade of transcriptional events coordinates the differentiation of pre-adipocytes into fully differentiated adipocytes; the process of adipogenesis. Although nicotinamide N-methyltransferase (NNMT) has been associated with obesity, how NNMT is regulated during adipogenesis, and the underlying regulatory mechanisms, remain undefined. In present study we used genetic and pharmacological approaches to elucidate the molecular signals driving NNMT activation and its role during adipogenesis. Firstly, we demonstrated that during the early phase of adipocyte differentiation NNMT is transactivated by CCAAT/Enhancer Binding Protein beta (CEBPB) in response to glucocorticoid (GC) induction. We found that *Nnmt* knockout, using CRISPR/Cas9 approach, impaired terminal adipogenesis by influencing the timing of cellular commitment and cell cycle exit during mitotic clonal expansion, as demonstrated by cell cycle analysis and RNA sequencing experiments. Biochemical and computational methods showed that a novel small molecule, called CC-410, stably binds to and highly specifically inhibits NNMT. CC-410 was, therefore, used to modulate protein activity during pre-adipocyte differentiation stages, demonstrating that, in line with the genetic approach, chemical inhibition of NNMT at the early stages of adipogenesis impairs terminal differentiation by deregulating the GC network. These congruent results conclusively demonstrate that NNMT is a key component of the GC-CEBP axis during the early stages of adipogenesis and could be a potential therapeutic target for both early-onset obesity and glucocorticoid-induced obesity.

## Introduction

Overweight and obesity are the fifth leading risk for global deaths, driven by comorbidities such as type 2 diabetes mellitus, cardiovascular disease, hypertension and stroke and certain forms of cancer^1^. Recently obesity has been associated with increased risk of hospitalization for COVID-19^2^. Biological, social and lifestyle determinants as well as the prolonged use of some medications such as glucocorticoids (GCs) can strongly contribute to adipose tissue dysfunction^3^. Obesity is a multifactorial disease caused by the energy imbalance occurring when intake is higher than consumption, promoting hyperplasia and/or hypertrophy^4^. Since mature adipocytes are postmitotic, hyperplasia requires a pool of precursor cells in the adipose tissue to differentiate into new adipocytes^5^.

Adipogenesis is a tightly coordinated program that integrates diverse extracellular signals within a temporally defined transcriptional network which can be divided into three phases: cell commitment, early- and terminal differentiation^4^. In vitro adipogenesis cell models are widely used, the 3T3-L1 cell line having become the gold standard. In post-confluent 3T3-L1 cell line, differentiation-inducing medium (DIM), composed of insulin, dexamethasone (DEX) and 3-isobutyl-1-methylxanthine (IBMX), activates IGF1 (Insulin Like Growth Factor 1), the glucocorticoid- and cAMP-signaling pathways, which in turn regulate the early differentiation stage. This adipogenic stimuli induces growth-arrested 3T3-L1 pre-adipocytes to synchronously reenter the cell cycle in a specific time window, the mitotic clonal expansion (MCE), when cells increase in number and activate a lineage commitment program, after which the cells permanently withdraw from the cell cycle, lose their fibroblast-like morphology and undergo terminal differentiation^6^.

One of the earliest, and critical, events that initiates this adipogenic cascade is the transcriptional activation of CEBPB, in fact, when over expressed in 3T3-L1 pre-adipocytes, it can initiate adipogenesis regardless of hormone stimulation^7^. Furthermore, MEFs from C/EBP (/) mice, as well as C/EBP knockdown in 3T3-L1 cells, do not undergo MCE and do not differentiate into adipocytes^8^. Although rapidly expressed 4 h post-induction, CEBPB lacks DNA-binding activity until the cells reenter the cell cycle, traverse the G_1_-S checkpoint, and initiate MCE, approximately 20 h after induction^9^. Having acquired its DNA binding activity, CEBPB activates the expression of several genes required for the differentiation process; among them the two late-acting adipogenic transcription factors *CEBPA* and peroxisome proliferator-activated receptor gamma (*PPARG*)^10^, which in turn initiate an autoregulatory and feed-forward circuit that allows the cells to establish an adipocyte identity that is maintained upon stimulation^11^. Since these two proteins also have anti-mitotic functions, their expression also coordinates the timing of MCE by closing this proliferative window^6^. Although the PPARG and CEBPA proteins have been widely recognized as core to the transcriptional network of adipogenesis, a large set of genes that play critical roles in fat cell differentiation has been identified^12–15^.

Nicotinamide N-methyltransferase (NNMT) is a metabolic enzyme that catalyzes the methylation of nicotinamide (NAM) using the universal methyl donor S-adenosyl methionine (SAM), linking the metabolic status of the cell with both methylation balance and intracellular levels of NAD+^16^. *NNMT* is abundantly expressed in liver and adipose tissue and in pathological conditions characterized by increased metabolic demand, such as some cancers, fatty liver disease and diabetes^17^. NNMT participates in several differentiation processes including naive-to-primed human embryonic stem cell transition^18^, myogenesis^19^ and both epithelial- and neural-mesenchymal transition in several cancers^20,21^. *NNMT* expression is regulated in a tissue and context specific manner rather than by a ubiquitous regulatory pattern^17^. Compelling evidence suggests a role for NNMT in obesity and type 2 diabetes^22^. *Nnmt* is upregulated in white adipose tissue (WAT) in a mouse model of obesity and diabetes^23^ and in the visceral adipose tissue of morbidly obese patients^24^. As a proof of concept, NNMT knock-down in the WAT and liver protected against diet-induced obesity, where it modifies the intracellular content of SAM and NAD+, two crucial mediators of cellular energy metabolism. In doing so, it controls the methyl donor balance, which in turn regulates the genes responsible for regulating energy consumption and promotes NAD+ depletion^23^. Additionally, some NNMT inhibitors (NNMTi) have been used to treat and/or prevent obesity and obesity-driven comorbidities in both preclinical animal models and 3T3-L1 cell line^25,26^. However, the underlying regulatory patterns and the mechanisms by which NNMT affects adipogenesis remain unclear. Using both a genetic and a pharmacological approach to suppress NNMT function, we demonstrated that NNMT is a key regulator of the early phase of adipocyte differentiation and that CEBPB regulates its expression following GC induction. Importantly, NNMT deficiency at this stage impairs the ability of pre-adipocyte’s to terminally differentiate.

## Materials and methods

### Cell culture and induction of differentiation

3T3-L1 Mouse Embryonic Fibroblasts were purchased from American Type Culture Collection (ATCC CL-173). The cells were propagated and maintained in Dulbecco’s modified Eagle’s medium (DMEM, Gibco) supplemented with 10% calf serum (FCS) at 37 °C in 5% CO_2_ atmosphere. Subconfluents cells were expanded and utilized for the subsequent experiments at the passages 3–6. To induce differentiation, 2-day post-confluent 3T3-L1 pre-adipocytes were cultured for 48 h with DIM (DMEM supplemented with 10% fetal bovine serum (FBS), 1.0 µM Dexamethasone (G. Biosciences), 1.5 mg/mL Insulin (Sigma) and 0.5 mM IBMX (Sigma). Two days after induction the DIM medium was removed and the cells were maintained in DMED supplemented with 10% FBS and 1.5 mg/mL Insulin, which was replaced every 48 h, until the cells became fully differentiated (8–10 days after induction). C3H10T1/2 cells were purchased from American Type Culture Collection (ATCC, clone 8, CCL-226, were grown and induced to differentiate following the same protocol, with cell cultures < 3 passages.

### RNA isolation and real-time PCR

Total RNA was extracted using the RNeasy kit (QIAGEN) and cDNA was synthesized by reverse transcribing 1 ug of total RNA using SuperScript™ III Reverse Transcriptase (Invitrogen) according to the manufacturer’s instructions. Real-time PCRs were performed using SYBR Green PCR Master Mix (Applied Biosystems) with an ABI PRISM 7900HT Sequence Detection System (Applied Biosystems). RT-PCR data were normalized to GAPDH and expressed relative to control (ddCt method). Real-time PCR primers are listed in Table 1.

**Table 1:** List of primers for *mus musculus* used in this study.

Primer name	Forward sequence (5′–3′)	Reverse sequence (5′–3′)
*Real-time PCR gene expression*		
*Gapdh*	GTGCAGTGCCAGCCTCGTCC	GCCACTGCAAATGGCAGCCC
*Nnmt*	CTTTGGGTCCAGACACTGTGCA	CCAGAGCCAATGTCAATCAGGAG
*Cebpb*	GGTTTCGGGACTTGATGCA	CAACAACCCCGCAGGAAC
*Cebpa*	TGGACAAGAACAGCAACGAGTAC	GCAGTTGCCCATGGCCTTGAC
*qPCR ChIP*		
CEBPB Binding Site 1 (CBS1)	AAATCACTCTGTAGTCCAGGCTGT	GACTCCAAAAGCTAACTTCACAGG
CEBPB Binding Site 2 (CBS2)	AGATTCCATCGTGTCTCAGCTC	TCTAAGACTCCAAAAGCCAACTTC
CEBPB Binding Site 3 (CBS3)	AAGTTGGCTTTTGGAGTCTTAGAG	ACGTCTGCAGTCTATTTCACTGTC
CEBPB Binding Site 4 (CBS4)	CAGTGAAATAGACTGCAGACGTTC	ACTCCCCTCTTCTCCTAAGCTC
CEBPB Binding Site 5 (CBS5)	TCATCTCCAAACAATCCAGTAGTC	TTGTTTGTTCAAACTGAAACCATT
CEBPB Binding Site 6 (CBS6)	AGAAAAGGGAATGTGGGGTAAG	CAGACTCCATCCGAGATTATTTTT
CEBPB Binding Site 7 (CBS7)	TTTAAAACATGGACATTGTCTTCC	CTCATTGAGTCCTAGTGGTGCTG
CEBPB Binding Site 8 (CBS8)	CTTCACAGAGTTGTTGCTCAAAAT	GGAGATTCTCTGGGTGAGTTTTTA
*Luciferase reporter vector*		
*Nnmt*-full promoter	CGATCTAAGTAAGCTGAGACGGGGTCTAACTATGTAACTG	CCGGAATGCCAAGCTCGTGTCTCAGCTCCGTGC
*Nnmt*-mut CBS4	CAGTGAAATAGACTGCAGACGTTCAGGCTACGTAAGACAGTGATGAAATTCCTGGAGAAGGGAGCTTA	TAAGCTCCCTTCTCCAGGAATTTCATCACTGTCTTACGTAGCCTGAACGTCTGCAGTCTATTTCACTG
*Nnmt*-mut CBS8	TTGATTAACCAGCCGGAGATGGGTAACACAATGCGAAAGCAGTAAGTTCCTGGCATCCCACG	CGTGGGATGCCAGGAACTTACTGCTTTCGCATTGTGTTACCCATCTCCGGCTGGTTAATCAA
*CRISPR gRNAs vector*		
*Nnmt*-Guide1	CTTGTGGAAAGGACGAAACACCG**GGAGAACTCCTGATTGACAT**GTTTTAGAGCTAGAAA	TTTCTAGCTCTAAAAC**ATGTCAATCAGGAGTTCTCC**CGGTGTTTCGTCCTTTCCACAAG
*Nnmt*- Guide 2	CTTGTGGAAAGGACGAAACACCG**GGGGACCAGTCAAAGGCTCC**GTTTTAGAGCTAGAAA	TTTCTAGCTCTAAAAC**GGAGCCTTTGACTGGTCCCC**CGGTGTTTCGTCCTTTCCACAAG
*Sanger sequencing*		
*Nnmt*-seq	CCTTCTTCAGCCATTTCTGC	TGCAGGTCCCTTCAGAAAGT

### Chromatin immunoprecipitation assay

Chromatin immunoprecipitation (ChIP) was performed following the instructions for SimpleChIP^®^ Enzymatic Chromatin IP Kit (Cell Signaling). For each immunoprecipitation performed, 1.6 × 10^6^ 3T3-L1 cells were seeded and grown to 2 days post–confluence (T0) before being treated, or not, with 1.0 uM DEX. After 20 h cells were fixed with 1% formaldehyde followed by glycine incubation. After cell lysis, chromatin was fragmented by micrococcal digestion and nuclear membranes were broken by sonication with a Bioruptor Sonicator (Diagenode). An aliquot of the digested chromatin (Input) was removed before sample immunoprecipitation with antibodies against either CEBPB (sc-7962, Santa Cruz Biotechnologies,) or a non-specific IgG control (Normal Rabbit IgG #2729, Cell Signaling). The complex co-precipitates were captured by Protein G magnetic beads and the cross-links were reverted before DNA purification. Chip-qPCR primers are listed in Table 1.

### Luciferase reporter assay

The promoter region of mouse *Nnmt* (− 1572 to − 1 bp) was amplified via PCR from genomic DNA extracted from 3T3-L1 cell line and cloned into pGL3 basic vector (kindly provided by Dr. Monica Álvarez). Full length *Nnmt* promoter (PGL3-Full Promoter) acted as template for site-directed mutagenesis to generate two mutant constructs (CBS4-mut and CBS8-mut) (primers are listed in Table 1), Sanger sequencing was performed to confirm the presence of the desired mutations. 80% confluent 3T3-L1 cells were transiently transfected with PGL3-Full Promoter, PGL3-CBS4mut, PGL3-CBS8mut and an empty vector along with a renilla luciferase pRL-CMV plasmid (kindly provided by Dr. Monica Álvarez) using Lipofectamine 3000 (Thermo Fisher) according to manufacturer’s instructions. Two-day post-confluent 3T3-L1 were treated with 1 µM DEX (DEX+) or medium alone (DEX−). 24 h after treatment the Dual-Luciferase Reporter Assay System (Promega) was used, according to the manufacturer’s instructions, to determine luciferase activity.

### Generating single cell-derived *Nnmt* knockout 3T3-L1 cell line

Two different single-guide RNAs (sgRNA) specifically targeting mouse *Nnmt* gene were designed using the CRISPR-ERA computational tool^27^ for gene repression. DNA oligonucleotides for the molecular cloning of the gRNA sequence (Table 1) were synthesized following the sequence structure 5-G-N19-NGG-3, as previously described^28^ adding a flanking region for proper cloning, using the In-Fusion^®^ HD Cloning Kit (Takara). These oligonucleotides were cloned in a BamH1-BsmB1 linearized pLenti-Guide-Puro vector (Origene, GE100032) using the In-Fusion^®^ HD Cloning system following the manufacturer’s recommendations. A two-vector approach was used to deliver Cas9 and the gRNAs to the cells. To assemble functional lentiviral particles, human HEK293 cells were independently transfected using Lipofectamine^®^ 3000 transfection reagents (Invitrogen) with lentiCRISPR v2 blast vector (Addgene, #83480), scramble pLenti-Guide-Puro vector (Origene, GE100032), and with the two pLenti-Guide-Puro vectors containing the specific gRNA sequences. The virus-containing medium was collected 48 h and 72 h after transfection, filtered with a 0.45-μm filter and used to transduce 3T3-L1 cell line. Two stable 3T3-L1 cell lines were obtained, after two weeks of antibiotic selection with puromicine (2 mg/mL) and blasticidine (5 mg/mL), by virally delivering CRISPRv2 blast with the two pLenti-Guide-Puro vectors, each carrying one sgRNA (3T3-L1-CRISPR-*Nnmt* Knockout) or with an empty pLenti-Guide-Puro vector (3T3-L1-CRISPR/CAS9 mock). Single cells were isolated and plated into a 96-well plate by single cell sorting (FACS), after which individual microcolonies were clonally expanded. Single cell-derived colonies were genotyped by Sanger sequencing and the resulting trace data were analyzed with Synthego^29^. Only those clones where the CAS9 cut generated a new stop codon in *Nnmt* sequences in both alleles were expanded and used for downstream applications.

### Oil red-O staining

Oil Red-O staining was used as previously described^30^. Briefly, cells were fixed for 30 min with 4% formaldehyde, stained with filtered Oil Red solution (0.2% oil red in 40% 2-propanol) and washed 5 times with distilled water. The accumulation of fat droplets in the cells was visualized and photographed under an inverted microscope (DMi1, Leica). The dye was eluted with 100% 2-propanol and quantified spectroscopically at 510 nm (Synergy HT, Bio-Tek).

### Flow cytometry cell cycle analysis

DNA staining with propidium iodide (PI) was used for cell cycle analysis. 3T3-L1 cells were collected at specific time points. For PI staining, cells were trypsinized, washed in PBS, fixed overnight with cold ethanol, stained with PI solution (3.8 mM sodium citrate, 50 µg/mL PI), and incubated with 0.5 mg/mL RNaseA for 45 min at 37 °C. DNA content was determined by FACS with a Cytomics FC500 Flow Cytometer (Beckman Coulter Life Sciences). Flow Cytometry data were analyzed with Modifit LT software (Verity Software House). Apoptosis was assessed using the Alexa Fluor^®^ 488 annexin V/Dead Cell Apoptosis Kit (Invitrogen, V13241 and V13245) following the manufacturer’s instructions, and the data were analyzed with CXP Flow Cytometry software (Beckman Coulter Inc).

### RNA sequencing and data analysis

RNA sequencing was performed at Genewiz (Azenta, Life Sciences) using a polyA selection protocol and the NEBNext ultra–RNA Library prep kit for Illumina. Sequencing libraries were clustered in an Illumina Novaseq instrument and sequenced using a 2 × 150 bp paired end (PE) configuration. On average, we obtained ~ 25 million PE reads per sample analyzed. Adapter removal from raw Fastq data was performed using Fastp (v_0.20.1)^31^. The subsequent filtered reads were aligned to the mouse GRCm38 genome using RSEM (v_1.3.1)^32^ and the hisat2-hca paired end mode. Mapping efficiency was above 75% in all samples analyzed. Estimated counts per gene were obtained using RSEM output and the tximport function of the tximport R/Bioconductor package (v_1.18.0)^33^. Differential expression analysis was performed using the R/Bioconductor package DESeq2 (v_1.30.1)^34^. To assess the statistical significance of differential expression changes between conditions in the context of a time course analysis (0 h, 20 h, 48 h) we adopted the likelihood ratio test (LRT) implemented in DESeq2. Our full model included the variables condition (mock or *Nnmt*-KO), time (0 h, 20 h, 48 h) and—the main variable of interest—the difference between the effects of conditions over time (interaction). The reduced model only included the variables condition and time. The resulting *p* values were corrected using the Benjamini and Hochberg method, considering a threshold of *p*.adj < 0.05 for statistical significance and we performed a rlog conversion of the data for downstream purposes. Differentially Expressed Genes (DEGs) (*p*.adj < 0.05) were clustered according to their gene expression profile along the time course of the experiment using rlog scaled values. Pairwise Spearman correlations, computed across the different profiles, were converted to distances and clustered using the hierarchical clustering approach (Ward.D method). The optimal number of clusters was determined using the elbow method that employs the within-cluster sum of square errors. Pathway enrichment of clusters was conducted using the Molecular Signature Database hallmark gene set collection^35^ and the R/Bioconductor package goseq (v_1.42.0)^36^, which accounts for multiple types of selection bias. As a control, all the genes identified in the RNAseq experiment were included as background universe. Molecular pathways with a *p*.adj < 0.05 were considered as significant for descriptive purposes. Raw RNA-Seq fastq files have been deposited at the European Nucleotide Archive (ENA PRJEB55900).

### Synthesis of CC-410

Methyl-(4*S*)-4-((3*S*,5*S*,7*S*,8*S*,10*R*,12*R*,13*S*,14*R*,17*S*)-3-acetoxy-7,12-diamino-10,13-dimethylhexadecahydro-1H-cyclopenta[a]phenanthren-17-yl) pentanoate -from now on termed as CC-410 for simplicity-was prepared in-house in multigram scale with a minimum of 98% purity following procedures previously described by our group^37,38^. The purity of the batches of compound CC-410 could be assessed by high-field nuclear magnetic resonance spectroscopy (^1^H and ^13^C-NMR) and high-resolution mass spectrometry. CC-410 was dissolved in dimethyl sulfoxide (DMSO; Sigma-Aldrich) and made up with the culture medium so that the concentration of DMSO was 0.5%. CC-410 molecule can be made available upon request at no cost.

### CC-410 cheminformatic evaluation

CC-410 chemical structure was initially submitted to the Open Innovation Drug Discovery program^39^ (Lilly Research Laboratories, Eli Lilly and Company, Indianapolis, USA), and it was selected for its novelty and drug-like properties, based on cheminformatic evaluation. Briefly, the submitted molecular structure was firstly processed based on the Lilly Medical Chemistry Rules, which are aimed at identifying and eventually excluding from screening those compounds that may interfere with biological assays. Next, structure-based filters, including MW, cLogP, solubility and toxicity among others were analyzed to select molecules with high druggable features. Additionally, the compound was also filtered based on the number of heavy atoms, number of rings, number of aromatic rings, ring size and other physicochemical properties. Solubility and permeability of the compound were also considered for the screening. For molecules passing the cheminformatic evaluation a substance check and comparison with known drugs (both Lilly and PubChem collections) was performed in order to select for novelty. Once the molecule passed this selection it was physically submitted to be tested with a high throughput screening study, querying the activity of the molecule against a wide variety of possible therapeutic targets. Among the tested targets, CC-410 showed exclusive inhibitory activity against human NNMT.

### NNMT inhibitor cell-based assay in HEK293 human hepatoma cell Line

Enzymatic and cell-based assays were performed by Lilly corporation (Lilly Research Laboratories, Eli Lilly and Company, Indianapolis, USA) and a full report with all the biological data was generated. Protocol from Lilly Research Laboratories: the HEK-293 (ATCC CRL-1573) cell line was seeded onto a 96-well plate at 25,000 cells/well in 0.1 mL growth medium and incubated at 37 °C in a 5% CO_2_/95% humidity incubator until 90% confluent. The day after, the 100× compounds were prepared in 100% DMSO in a 96-well polypropylene storage plate, starting from 10 mM for 11-points at 1:3 serial dilutions. 2.5 mL of 100× compounds were diluted onto fresh 96-well polypropylene plates containing 97.5 mL/well of growth medium to make 2.5×, then were transferred to 80 mL/well of 2.5× compound onto cell plate and incubated for 2 h at 37 °C in a 5% CO_2_/95% humidity incubator. 10× substrate mix were prepared in growth medium: 0.25 mM SAM (Sigma #A7007) and 5 mM NAM (Fluka #72340) or NAM-d_4_ (CDN Isotopes #D-3457). Followed by the addition of 20 mL/well of 10X substrates mix onto cell plates and incubated overnight at 37 °C in a 5% CO_2_/95% humidity incubator. 24 h post addition of substrate, 0.1 ml/well of conditioned medium was transferred onto 96-well polypropylene storage plates for measuring SAM, NAM, SAH, and MNAM or MNAM-d_4_ by LC-MS.

### Automated solid-phase extraction and mass spectrometry

An Agilent 300 RapidFire automated extraction system (Agilent, Santa Clara, CA) with three HPLC quaternary pumps was coupled to an Agilent 6495 triple quadrupole mass spectrometer (Agilent Technologies, Santa Clara, CA) equipped with an electrospray ionization (ESI) interface source. The RapidFire Mass Spec system was equipped with a reusable HILIC (type H1) solid-phase extraction (SPE) cartridge (G9209A). Solvent A, used for sample loading and washing, was 0.1% formic acid in acetonitrile. Solvent B, used for sample elution, was 50% acetonitrile containing 0.1% formic acid. Samples were sequentially analyzed by aspirating 10 μL onto the collection loop under vacuum directly from multiwell plates. The 10 μL of sample was loaded onto the HILIC cartridge and washed, by quaternary pump 1, using solvent A at a flow rate of 1.25 mL/min for 6000 ms. The retained analytes were then eluted to the mass spectrometer by quaternary pump 3, using solvent B at a flow rate of 1.25 mL/min for 6000 ms. The system was re-equilibrated by quaternary pump 1, using solvent A at a flow rate of 1.25 mL/min for 3000 ms. The triple quadrupole mass spectrometer was equipped with an electrospray ionization (ESI) source and analytes monitored using selected reaction monitoring (SRM) in positive mode [M-H]+. S-Adenosyl-L-homocysteine was monitored at m/z 385.1/136.0, 13C10 S-Adenosyl-L-homocysteine was monitored at m/z 395.1/141.1. Methyl nicotinamide was monitored at m/z 137.1/94.0 and D7 methyl nicotinamide was monitored at m/z 144.1/101.1. The area ratio values for each metabolite were calculated using their respective internal standard. The IC_50_ values were determined by fitting the inhibition curve (percentage inhibition versus inhibitor concentration) using a four-parameter logistic curve. Values are calculated by dividing the AUC of the product (MNAM or SAH) by the AUC of their internal standard (d7-MNAM and d10-SAH, respectively). Data are area ratio values for MNAM and SAH.

### Methyltransferases hot spot assay

CC-410 was screened in biochemical assays for activity against a panel of 11 methyltransferases (MT) by using the radioisotope (3H-AdoMet) based MT Hot Spot Assay. All the assays were performed by Reaction Biology Corporation (RBC, Malven, PA, USA). Briefly CC-410 was incubated with the selected MTs, corresponding substrates, and radiolabeled SAM (S-Adenosyl-L-[methyl-3 H]methionine). The specific MTs assessed, and the corresponding substrates are as follows: DNMT1- Poly dl-dC, DNMT3a- Lambda DNA, DNMT3b- Lambda DNA, DOT1L- Nucleosomes, EZH2 Complex- Core Histone, METTL21A- HSPA8-[CTD], NRMT1- RCC1, PRMT3- Histone H4, PRMT5/MEP50- Histone H2A, SET1b -Core Histone Complex and SMYD2- Nucleosomes. Reaction mixtures were incubated and spotted onto filter papers, which were then washed to remove unreacted SAM, leaving the bound radiolabeled product. Detection of the radiolabeled, methylated product was performed via a nanoliter-scale radioisotope filter binding platform. The CC-410 compound was tested in 10-dose IC_50_ mode with threefold serial dilution, in singlet, starting at 300 μM. For each assay, control compounds were included as positive controls for enzyme function and assay reproducibility. Control compound, SAH (S- (5′-Adenosyl)-L-homocysteine), or LLY507, (used for SMYD2 assay) were tested in 10-dose IC_50_ mode with threefold serial dilution starting at 100 μM. IC_50_ was calculated by non-linear least square fitting dose–response curve. Percentage of enzyme activity (no inhibitor control as 100% activity) and curve fits were generated for all the control compounds and where the enzyme activities at the highest concentration of CC-410 were less than 65%.

### Docking computation in hNNMT a mNNMT and molecular dynamics

The structure of CC-410 ligand was computed, in vacuum, at the b3lyp/6-31+G** level of theory. The so obtained xyz coordinates were used for docking study. Coordinates for (*S*)-4-((((2*R*,3*R*,4*R*,5*R*)-5-(6-amino-9H-purin-9-yl)-3,4-dihydroxytetrahydrofuran-2-yl)methyl)(naphthalen-2-ylmethyl)amino)-2-ammoniobutanoate a.k.a. compound 8^40^ was generated with the aid of Chemoffice 2019 (Perkin Elmer) and UCSF Chimera^41^. Docking calculations were carried out with AutoDock Vina^42^ using a 25 Å × 25 Å × 25 Å region of interest targeting the center of mass of the active-site cavity. Recombinant mNNMT atomic coordinates—PDB ID: 5YJI^25^ and hNNMT, PDB ID: 6b1A^43^ and 2IIP (Bernstein et al., unpublished) were used as CC-410 targets. Notably, 5YJI coordinates already include a molecule of S-adenosyl-homocysteine (SAH) as ligand in the active site pocket. The hNNMT and mNNMT structures also incorporate coordinates of an His_6_ tag and a thrombin cleavage site at the interface between distinct biological molecules. These residues from the tag and the cleavage site have not been considered in the docking computations. Residues missing in the structure because of their mobility were added with Modeller 9v7^44^. The resulting zDOPE score was − 2.23. The structure of mNNMT in complex with bisubstrate inhibitor MS2756^43^ was used for comparison.

### Molecular dynamics and structural analysis

Molecular dynamics (MD) computations were performed with the PMEMD module of AMBER 16^45,46^ under the AMBER-14SB force field^47^ for the protein, and the General Amber FF^45^ for the ligand atoms. The amine groups of CC-410 were protonated as pK_a_ estimations yielded values above 9.5. AM1-BCC atomic charges^48^ for CC-410 were computed with the Antechamber module of AMBER. Apo-proteins and ligand target complexes were simulated under periodic boundary conditions using orthorhombic cells. The minimum distance between protein and cell faces was 10 Å. Sodium counter-ions were added to neutralize the system. The structures were solvated using the SPC/E water model^49^. Particle Mesh Ewald (PME) electrostatics were set with the Ewald summation cut-off at 9 Å. The SHAKE algorithm^50^ served to constrain bonds involving hydrogen atoms. The whole system was subjected to energy minimization, then to 500 ps of NPT MD to adjust box size and solvent density, followed by additional energy minimization and equilibration at 298 K using a Langevin thermostat. Then, 100 ns of production trajectories were recorded. These were analyzed with CPPTRAJ^51^. CASTp analysis^52^ enabled cavity characterization. LigPlot^53^ was used to explore protein–ligand interactions.

### Cell viability assay

CellTiter-Blue^®^ Cell Viability Assay (Promega) was used following the manufacturer’s instructions. 3T3-L1 cells were seeded at a density of 2 × 10^3^ cells per well in 96-well plates and cultured till confluence in standard medium. Two-day post-confluent 3T3-L1 cells were induced with DIM and treated with 1, 5, 10 and 25 µM CC-410 along with an untreated control (0.5% DMSO) in 4 different conditions: every 48 h throughout the differentiation period; once in the first 20 h following DIM stimulation; once 48 h after DIM stimulation; or after the early differentiation phase. In all cases, cell viability was assessed at terminal differentiation. Cell viability percentages were calculated by normalizing the fluorescence recorder (560/590) for the treated samples over untreated controls.

## Results

### Glucocorticoids induce CEBPB-mediated transactivation of Nnmt during the early phase of 3T3-L1 pre-adipocytes differentiation

**Figure 1:**
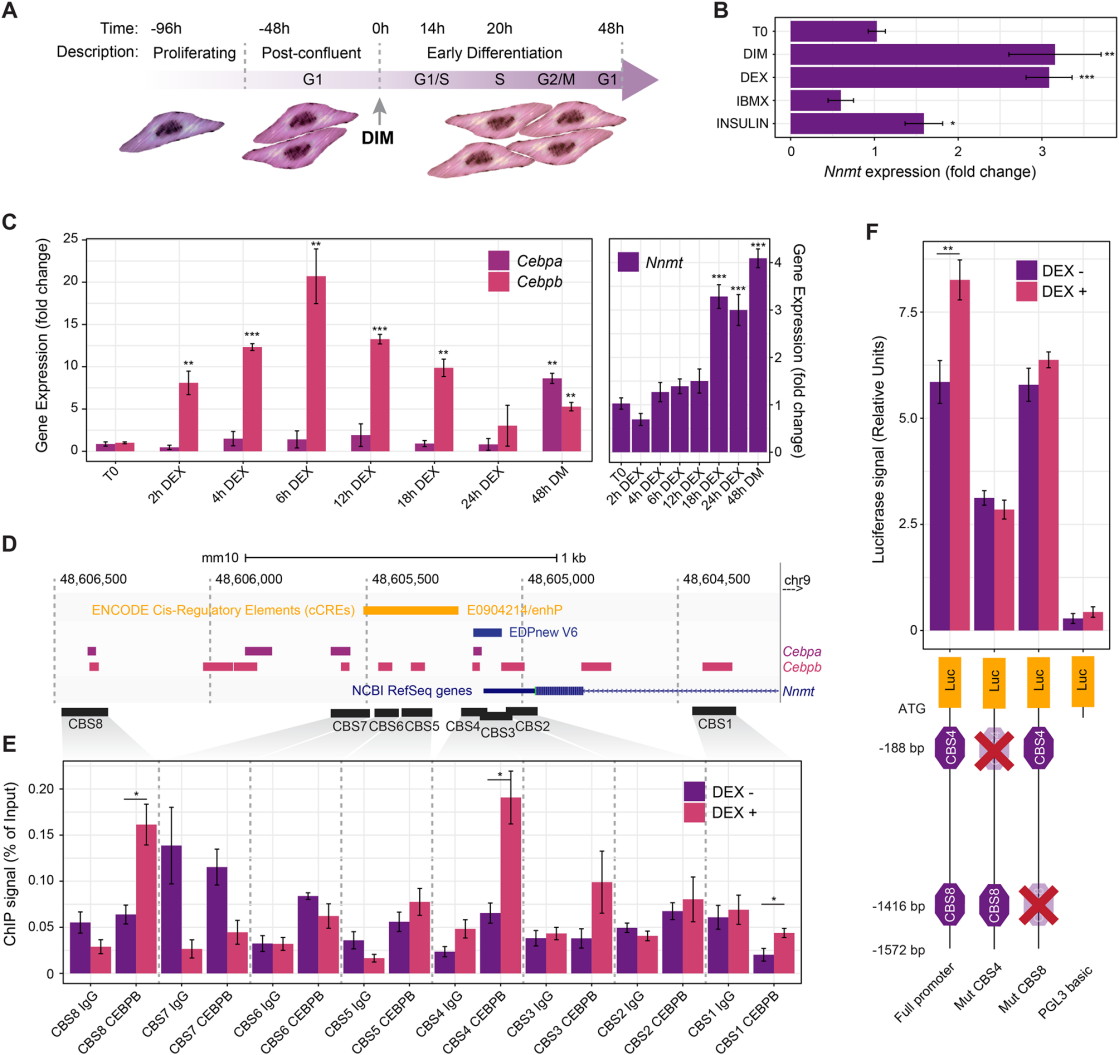
Transactivation of Nnmt by CEBPb is regulated by dexamethasone during the early phase of adipogenesis. (**A**) Schematic representation of 3T3-L1 pre-adipocyte differentiation protocol and chronological progression through the different phases of the cell cycle during the early phase of adipogenesis. (**B**) mRNA levels of *Nnmt* gene measured by Real-Time PCR after post confluent 3T3-L1 cells (T0) were exposed for 48 h to individual components of the DIM alone or in combination. Values are expressed as fold change (mean ± S.D, n = 3) relative to the untreated control (T0). (**C**) After DEX treatment, mRNA levels of *Cebpb*, *Cebpa* and *Nnmt* were analyzed at the times indicated by RT-qPCR. Data were normalized to the T0 time point of each gene. (**D**) CEBPB and CEBPA binding sites in the proximal and distal region of *Nnmt* promoter, obtained from the GTRD database, also including surrounding ENCODE cis- regulatory elements. Black rectangles represent the 8 putative CEBPB binding sites (CBS), corresponding to the amplicons spanning the ChIP-qPCR primer-targeted regions. (**E**) Effect of DEX on CEBPB recruitment to the *Nnmt* promoter. Post-confluent 3T3-L1 cells were treated, or not, with DEX (1uM) for 20 h and CEBPB binding on *Nnmt* promoter was analyzed by Chip-qPCR, with primer pairs specific to the putative CEBPB binding sites. Chip-qPCR was performed using control IgG and anti-CEBPB antibody. Chip data were expressed as percentage of input normalized to control IgG. Statistical significance was calculated by means of a one-sided Welch’s t-test between DEX- and DEX+ conditions for each amplicon. For interpretation purposes, only the regions that showed enrichment with anti-CEBPB higher than the control IgG for the same sets of primers were considered for the final statistical assignments. (**F**) 3T3-L1 cells were transfected with reporter plasmids containing the full *Nnmt* promoter (~ 1500 bp) or two mutant constructs (mut CBS4 and mut CBS8) and grown two days post-confluence before treatment, or not, with DEX for 20 h. Firefly and renilla luciferase activity were measured sequentially and the results expressed as ratios of firefly luciferase activity over relative renilla luciferase activity. An empty reporter vector was also used as internal control. The data represent three independent experiments, each with at least three technical replicates, and values are expressed as mean ± SEM. Statistical significance was calculated by means of a one-sided Welch’s t-test. For all the panels: *p < 0.05; **p < 0.01; ***p < 0.001.

To address the molecular and temporal regulation of NNMT, we used the standard protocol for inducing and tracking the synchronous progression of 3T3-L1 pre-adipocytes towards terminally differentiated adipocytes (Fig. 1A). Since these cells irreversibly commit to differentiating during the early phase of adipogenesis, we sought to investigate *Nnmt* expression and its regulatory pathways during this period. Confluent 3T3-L1 cells were treated for 48 h with DIM or individually with each component of the induction cocktail. Quantitative RT-PCR revealed that *Nnmt* expression increased after hormonal stimulation, and that DEX produced induction comparable with that of the whole cocktail. However, neither insulin nor IBMX impacted *Nnmt* expression significantly compared to unstimulated cells (Fig. 1B), demonstrating that NNMT is activated during the early phase of adipogenesis mainly by glucocorticoid signaling. Because of the well-recognized and temporally defined role of CEBPA and CEBPB during adipocyte differentiation, we followed their expression after DEX treatment. In agreement with previous reports^54,55^, DEX itself is able to induce a rapid increase, as soon as 2 h after induction, in *Cebpb* expression, which peaked between 4 and 12 h post-induction and gradually declined to almost its basal level at 24 h (Fig. 1C). As expected, *Cebpa* induction started later, almost 48 h after DEX induction, once the cells start withdrawing from the cell cycle. It is worth noting that *Nnmt* expression increased 18–24 h after DEX treatment, synchronously with the acquisition of CEBPB DNA binding activity and prior to *Cebpa* induction (Fig. 1C), suggesting that *Nnmt* could be a CEBPB transcriptional target. To study the binding of CEBPB to *Nnmt* promoter, we performed Chip-qPCR on both differentiating cells harvested 20 h after DEX treatment and unstimulated controls. We extrapolated putative CEBPB binding sites in the proximal and distal region of *Nnmt* promoter using the Gene Transcription Regulation Database (GTRD)^56^ (Fig. 1D). Experimental validation by Chip-qPCR confirmed that two regions, referred as CBS (CEBPB Binding Site) 4 and CBS 8, spanning respectively the chromosomal positions chr9:48605113-48605195 and chr9:48606327-48606478, were significantly enriched by CEBPB antibody 20 h after DEX induction (Fig. 1E) but not in the unstimulated samples, indicating that CEBPB binds to *Nnmt* promoter following GC treatment. The proximal CEBPB regulatory region CBS4 was located near a CIS-regulatory element and overlapped with the *Nnmt* transcription start site reported by eukaryotic promoter database (EPD)^57^ (Fig. 1D). Previous research^21^ demonstrated that in glioblastoma (GBM), CEBPB is the transcription factor that most significantly correlates with Nnmt expression, and that binding of CEBPB to Nnmt regulatory regions upregulates its expression. It is worth noting that the CBS8, discovered by ChIP-PCR in our study, overlaps with the most significantly CEBPB enriched regulatory motif on the NNMT promoter, previously characterized in GBM. These data suggest that both proximal CBS4 and distal CBS8 could be *bona fide* CEBPB regulatory elements. This was further confirmed by mutating the CEBPB consensus sequences within the CBS4 and CBS8 regions. A DNA *wild-type* fragment containing the 5′-flanking region of the *Nnmt* gene (from 0 to 1572 bp) and the two mutated constructs (Mut CBS4 and CBS8) were subcloned in a PGL3-basic plasmid and transfected in post-confluent 3T3-L1 treated or not with DEX for 20 h. Notably, hormonal stimulation in cells transfected with wild-type *Nnmt* promoter-luciferase plasmid caused a statistically significant increase in luciferase activity. Contrarily, mutations disrupting the consensus sequences for the two potential CEBPB binding sites totally abolished this transactivation (Fig. 1F). Overall, these data shows NNMT is activated during the early phase of adipogenesis through the GC transcriptional network and its transactivation is mediated by CEBPB recruitment onto the Nnmt promoter region.

### *Nnmt* ablation suppresses terminal adipocyte differentiation by delaying cell cycle exit during MCE

**Figure 2:**
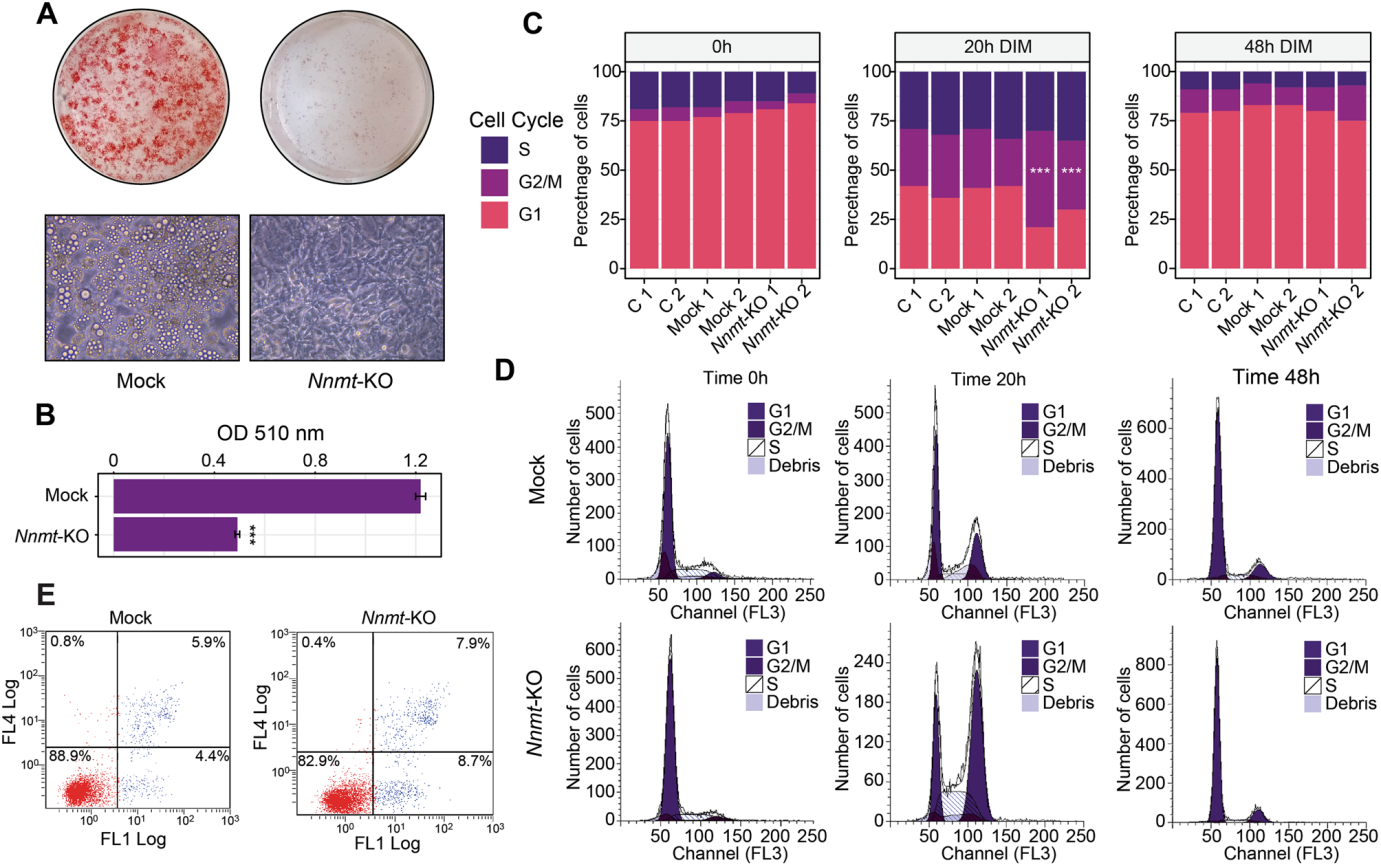
Nnmt deficiency impairs adipocyte differentiation by regulating MCE. (**A**) Terminal adipogenesis was analyzed 10 days after DIM induction in 3T3-L1-*Nnmt*-KO (3T3-L1-CRISPR/CAS9 *Nnmt* guides) and mock control cells (3T3-L1-CRISPR/CAS9 empty) by Oil Red-O staining and bright field microscopy (20× magnification). (**B**) Statistical data for Red-Oil staining quantification by OD measurements. Data are presented as means ± S.D. (n = 3) and statistical significance was calculated with a one-sided Welch’s t-test. (**C**) DNA content was analyzed by PI staining and FACS analysis was performed at different times after hormonal stimulation (T0, 20 h DIM and 48 h DIM). The percentage of cells in each phase of the cell cycle are represented in the graph plot, which shows the results of three independent experiments. Statistical significance was calculated by a chi-squared test using a 3 × 2 contingency table including the percentage of cells at each cell cycle stage (G1, S, G2/M) and in each experimental condition (mock – *Nnmt*-KO) per triplicate. (**D**) Flow cytometry plots representative of one independent experiment. (**E**) Annexin and PI staining. *Nnmt*-KO and mock cell lines were stained 48 h after DIM induction with Fluor 488 annexin V and PI and analyzed by flow cytometry. The loss of *Nnmt* did not influence either cell viability or apoptotic rate. For all the panels: *p < 0.05; **p < 0.01; ***p < 0.001.

**Figure 3:**
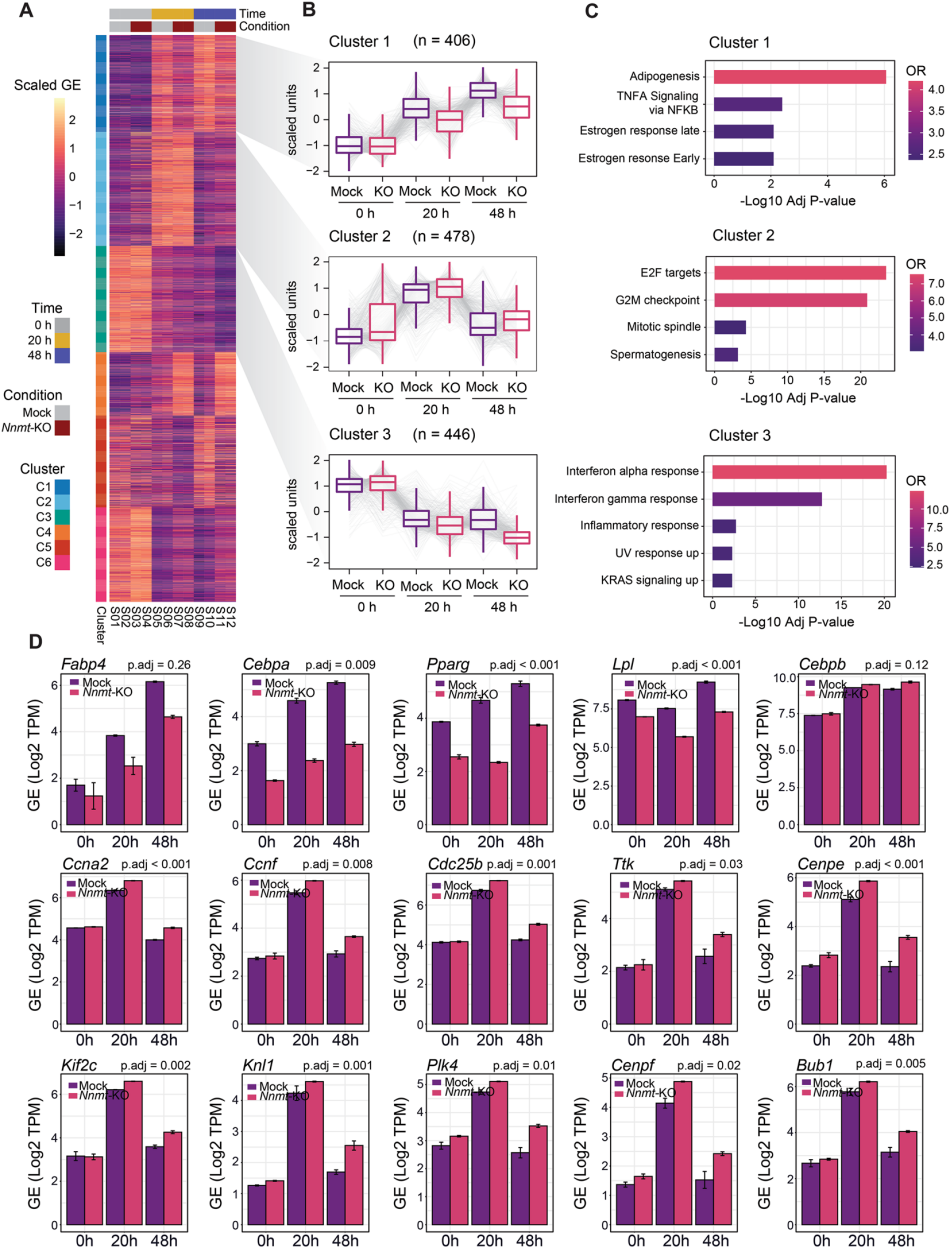
NNMT regulates molecular pathways related with fat cell differentiation and cell cycle progression. (**A**) Heatmap representing the gene expression values of the significant genes (Adj. *p*.value < 0.05) obtained from a likelihood ratio test analysis comparing *Nnmt*-KO and mock samples across different time points. Genes were ascribed to different clusters according to their expression pattern. (**B**) Boxplots indicating the scaled gene expression values observed for genes contained in clusters 1, 2 and 3. (**C**) Barplots depicting the significant enrichment of molecular signatures (Hallmark gene set, MSigDB) of clusters 1, 2 and 3. Bar length represents statistical significance (− Log10 Adj. *p*.value) and color scale represents the Odds Ratio of the different gene pathways interrogated. (**D**) Differentially expressed genes in *Nnmt*-KO compared to mock 3T3-L1 cell lines during MCE as determined by RNA sequencing. The upper panel shows selected genes known to have a key role in adipogenesis. The middle panel shows the time-dependent upregulation of G2/M transition-related genes. And the lower panel shows selected genes related to cytoskeleton organization during cell cycle cytokinesis. For all the plots, gene levels are represented as the log2 of the Transcripts Per Kilobase Million (TPM), and statistical significance for the conditions was assessed using a likelihood ratio test, as described in the methods section.

To investigate how NNMT regulates adipogenesis, we generated stable, single cell-derived *Nnmt* knock-out 3T3-L1 cell lines (*Nnmt-KO*) with the CRISPR-Cas9 system^28^. Knockout efficiency was confirmed by Sanger sequencing and gene expression analysis. The selected *Nnmt-KO* cell line was cultured to confluence and induced to differentiate for 10 days. Oil Red-O staining showed that *Nnmt-KO* accumulated significantly less lipid than the control cell line (Fig. 2A,B). Morphological inspection confirmed that *Nnmt* ablation induced a severe adipogenesis defect and locked the cells in a pre-differentiated fibroblast-like stage, suggesting that NNMT is required for adipogenesis through regulating the adipose lineage commitment stage. Since we previously demonstrated that *Nnmt* is activated during MCE, we investigated whether NNMT deficiency could affect cell cycle progression during this time interval. Flow cytometry analyses revealed that *Nnmt* depletion did not affect cellular distribution in normally proliferating (data not shown) or post-confluent cell line (T0) (Fig. 2C,D). As expected, hormonal stimulation induced the growth-arrested cells to reenter the cell cycle, although 20 h post-induction *Nnmt-KO* resulted in a significant increase in cell population in G2/M phase (from 27 to 42%) with a concomitant reduction in G1 phase (from 41 to 25%) (Fig. 2C,D). To ascertain whether *Nnmt* inactivation produces a permanent or a transient block in the G2/M phase, FACS analysis was performed 48 h after DIM induction. Neither statistically significant differences in cell cycle distribution (Fig. 2C,D) nor increases in the percentage of apoptotic cells (Fig. 2E) were observed between control and *Nnmt*-KO cell lines, suggesting that, rather than a permanent block, lack of *Nnmt* induces a delay in the cell cycle progression during MCE. A recent study on adipogenic cell model demonstrated that cells undergoing terminal differentiation exit the last mitosis early compared with cells that do not differentiate^58^. Considering this, we can assume that NNMT controls pre-adipocyte commitment by coordinating the proper progression through the cell cycle during MCE. To fully understand the molecular mechanisms underlying NNMT function during adipogenesis, we performed gene expression profiling at different time points during MCE. RNA sequencing experiments were carried out on *Nnmt*-KO and mock control cell lines harvested at T0 (growth-arrested pre-adipocytes), 20 h (during MCE) and 48 h (post-mitotic) after DIM stimulation. To identify genes influenced by *Nnmt* depletion along this time course, we performed a Likelihood ratio test followed by a classical gene clustering approach. This strategy led to the identification of six enriched gene clusters (Fig. 3A). Cluster 1 was enriched in genes that showed progressive upregulation as a direct consequence of hormonal stimulation and are assumed to have a leading role in the differentiation process (Fig. 3A,B). Consistent with *Nnmt-KO* phenotype, adipogenesis was among the strongest enriched pathways in this cluster (Fig. 3C). At T0, the genes from this cluster were downregulated overall, with no statistically significant differences between *Nnmt-KO* and control cells lines. Interestingly, already at 20 h and more pronounced 48 h after hormonal stimulation, we observed an upregulation of adipogenesis-related genes in the control cell line that was significantly lower in the knock-out cell line, confirming that NNMT acts in a clearly defined period during the early phase of adipogenesis. As evidence, *Nnmt* depletion was associated with a reduction in the mRNA level of late-acting adipogenic genes such as, *Cebpa*, *Pparg* and *Lpl* (Lipoprotein Lipase), but did not influence *Cebpb* expression (Fig. 3D), corroborating that it may act upstream of NNMT by regulating its expression. Cluster 2 was significantly enriched in genes related to cell cycle progression, particularly E2F, the G2/M checkpoint and the mitotic spindle assembly pathways (Fig. 3B,C). The expression of these genes fluctuated in line with cell cycle phases during MCE and with FACS analysis (Fig. 2C,D). In fact, the expression of cluster 2 genes peaked 20 h after hormonal stimulation, indicating that the cells actively re-entered the cell cycle, and then dropped to a level comparable with T0 as the cells permanently withdrew from the cycle after MCE (48 h). However, compared to control cell line, *Nnmt-*KO induced an overall delay in downregulating G2/M-related genes, confirming that *Nnmt* depletion retains a higher population of cells in this cell cycle phase and that it can regulate cell commitment by shifting the timing of last mitotic exit. Of note, we found that *Nnmt* deficiency influence key factors in G2-M transition as well as genes involved in cytoskeleton organization during cytokinesis (Fig. 3D). Cluster 3 was enriched in genes belonging to the interferon-alpha and interferon-gamma signaling pathways (Fig. 3B,C). In accordance with their anti-adipogenetic function^59,60^, these two pathways showed an overall downregulation after hormonal stimulation, with statistically significant differences between the KO and control cell lines. Hypoxia and apical junctions and epithelial to mesenchymal transition were among the other significantly enriched pathways identified, indicating the potential role of NNMT as a central metabolic regulator of these cellular processes.

### CC-410 is a new efficient and specific small molecule inhibitor of NNMT

**Figure 4:**
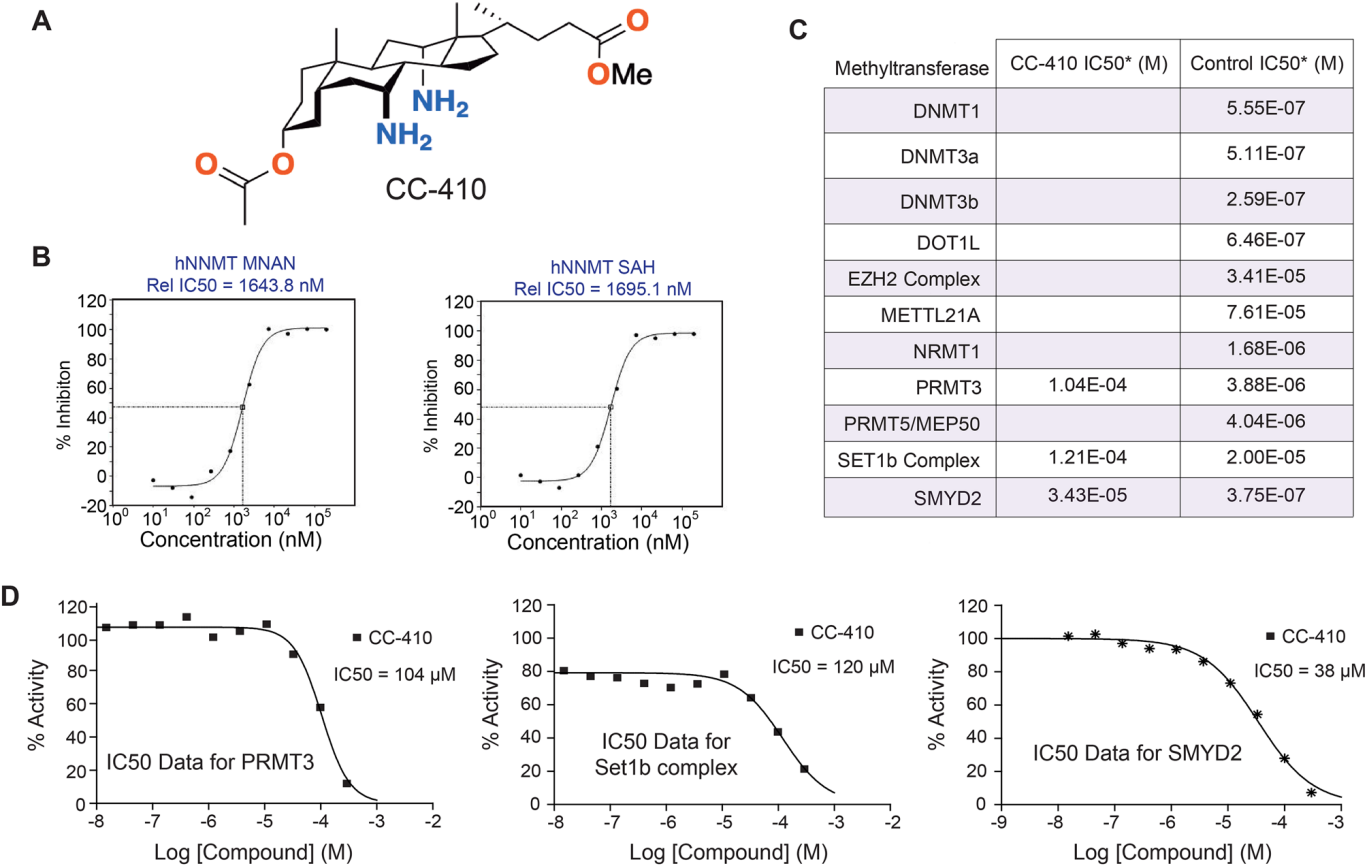
CC-410 specifically inhibits NNMT. (**A**) The chemical structure of CC-410 compound. (**B**) concentration response curve (CRC) of hNNMT enzymatic modulation using LC/MS assay (IC_50_). The area ratio values for each metabolite were calculated using their respective internal standard. (**C**) Activity of CC-410 against 11 methyltransferases. Summary of IC_50_ values for CC-410 and respective controls. CC-410 compound was assessed in 10-dose IC_50_ mode with threefold serial dilution, starting at 300 μM. Control compound, SAH (S-(5′-Adenosyl)-L-homocysteine), or LLY507, was tested in 10-dose IC_50_ mode with threefold serial dilution starting at 100 μM. The empty cells indicate no inhibition or compound activity that could not be fitted to an IC_50_ curve. (**D**) Percentage of enzyme activity and curve fits for the only three methyltransferases that were moderately inhibited by CC-410. Curve fits were performed where the enzyme activity at the highest concentration of compounds were less than 65%.

**Figure 5:**
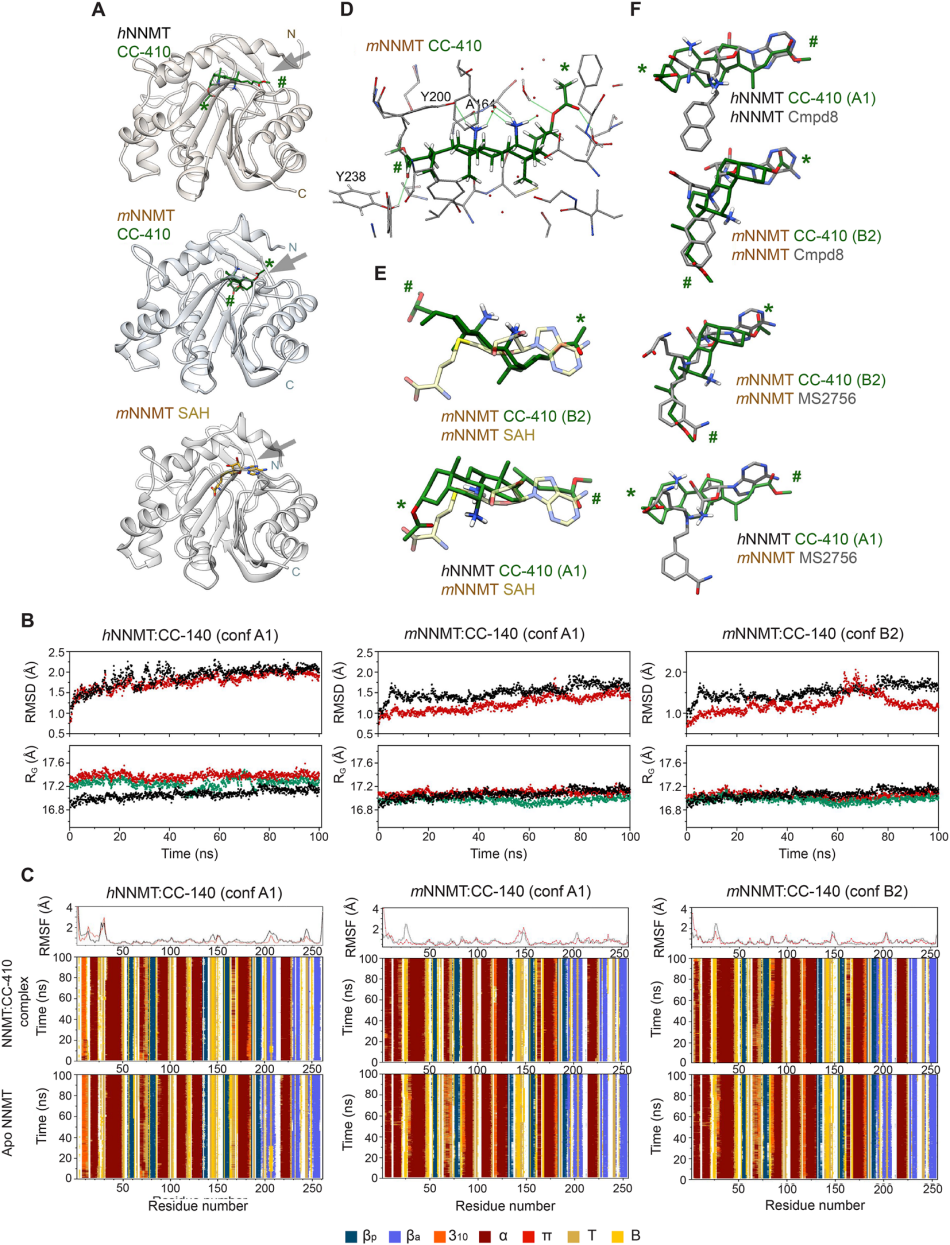
Molecular docking and molecular dynamics simulation reveal that CC-410 specifically inhibits NNMT by stably binding to its catalytic active site. (**A**) Best solutions of docking computations of CC-410 targeting *h*NNMT (top) and *m*NNMT (middle), and the complex between *m*NNMT and SAH (bottom) according to the X-ray diffraction model (PDB 5YJI). All the structures are based on the coordinates closest to the average of the last 70 ns of 100 ns molecular dynamics (MD) trajectories. Protein backbones are represented by Richardson’s ribbon diagram and the CC-410 molecule by sticks. Carbon atoms are in green, nitrogen in blue, oxygen in red and sulfur in yellow. Gray arrows point to the active-site cavity opening. (**B**) Statistics of MD computations. For each set of computations, the upper panel shows the Root Mean Square Deviation (RMSD) of protein main chain atoms with respect to the energy-minimized structure throughout trajectory calculations in the presence (red) and absence (black) of CC-410 ligand. The lower panels show radii of gyration (*R*_G_) of NNMT in the presence and absence of ligand. In red, *R*_G_ of NNMT protein moiety in the presence of ligand, in green, *R*_G_ of NNMT plus ligand, in black, *R*_G_ of NNMT along the trajectory without ligand. (**C**) Atomic fluctuations and secondary structure analysis. Upper panels*:* Backbone fluctuations represented as per-residue root mean square fluctuations (RMSF) of atomic positions with respect to their average. Data corresponding to the apoprotein are in black, those for the bound protein in red. Lower panels*:* timeline of secondary structured as determined with Kabsh’s algorithm. Extended conformations are in blue, α-helices in dark red, 3_10_ helices in orange, p helices are in red, turns are in beige, and bends in yellow. (**D**) Details of the structure closest to the MD average of CC-410 bound to *m*NNMT. Green lines represent hydrogen bonds. (**E**) Structural alignment of the MD-refined docking results of CC-410, in the B2 (mNNMT) and A1 (*h*NNMT) orientations, with SAH. Only the ligands are shown. (**F**) Structural alignments of bifunctional drugs MS2756 and compound 8 (cmpd8) docked to *m*NNMT with SAH and CC-410 docking solutions. The view has been rotated arbitrarily with respect to panel C to highlight the moiety of the bifunctional ligands that enter the nicotinamide (methyl-acceptor) pocket. * and ^#^ stand for R1 and R2 sidechains, respectively.

Small molecules are useful chemical tools that allow the modulation of protein function in a tunable and conditional manner. To this end, we characterized by biochemical and computational methods a novel specific small molecule inhibitor of NNMT, called CC-410. CC-410 was previously synthetized by our group ^37,38^; the molecule belongs to the steroid family, natural products with structural, regulatory, and hormonal functions. Particularly, CC-410 derives from cholic acid, a cheap naturally-occurring bile acid produced by the liver of mammals. It is equipped with two primary amine groups, borne on the C7 and C12 positions of the steroidal scaffold, and an ester function. These groups define a densely-functionalized cavity capable of hydrogen bonding and other supramolecular interactions (Fig. 4A). CC-410 was initially submitted to the Lilly Open Innovation Drug Discovery program ^39^ (Lilly Research Laboratories, Eli Lilly and Company, Indianapolis, USA). Based on its cheminformatics evaluation, it was privileged for its drug-like properties and its activity was assessed against a wide variety of possible therapeutic targets, showing exclusive inhibitory activity against human NNMT. The concentration-dependent activity of CC-410 against human NNMT was evaluated using automated solid-phase extraction and liquid chromatography mass-spectrometry (LC–MS), resulting in an IC_50_ of 1.6 µM (Fig. 4B). The selectivity of CC-410 was further assessed by biochemical assays for activity against a panel of 11 MTs, chosen according for their structural and sequence similarities or because they have been described as possible off-targets of other NNMTi molecules ^26,43^. In the range of concentrations tested, CC-410 did not influence the activity of most MTs (Fig. 4C). In fact, of the 11 enzymes examined, only 3 MTs were inhibited by CC-410, although with IC_50_ values far greater than those obtained against NNMT. Specifically, CC-410 slightly inhibited PRMT3 and SET1b Complex (IC_50_ of 104 and 120 µM, respectively), while IC_50_ for SMYD2 was 34.25 µM (Fig. 4D). The fact that SMYD2 is not expressed in adipose tissue ^61^ excludes the possibility that, at least in this context, this MT might represent a possible off-target of CC-410. Computational docking studies were furthermore performed to elucidate how CC-410 interacts with the active sites of human (*h*NNMT) and mouse (*m*NNMT) NNMT proteins (Fig. 5A). CASTp analysis ^52^ of *h*NNMT (PBD-ID: 6B1A) and *m*NNMT (PBD-ID:5YJI) structures revealed a highly conserved internal cavity which includes residues necessary for catalysis, including a methyl-donor site interconnected to an acceptor site. Notably, CC-410 molecules have a polycyclic sheet structure with two different faces, amino groups on one and methyl groups on the other, and two different sidechains, with the acetoxi- (R1) and 5-methoxy-5oxopentan-2-yl (R2) at opposing ends (Fig. 4A). In line with this, best docking results (Table 2) showed that CC-410 well fits the NNMT cavity in two major orientations. In the first (A1), the R2 of CC-410 is located near the cavity opening while the rest of the inhibitor, including R1, completely fills the donor pocket of the active site. In the second orientation (B2) the R1 is located near the cavity opening and the R2 group lies within the acceptor pocket and the inhibitor partially occupies the donor site. The comparison of evaluation scores suggested that these alternative orientations are similarly stable, although best results for *h*NNMT had A-type orientation, whereas *m*NNMT preferred the B orientation. Since simulations yielded better scores with *m*NNMT than *h*NNMT, CC-410 was again docked with another set of *h*NNMT coordinates (PDB ID: 2IIP), confirming that the preference of *h*NNMT for A conformations was independent of the model chosen (Table 2). Best docking results were subjected to MD computations, including control trajectories without the ligand (Table 3). Along the trajectories, the structures of both *h*NNMT and *m*NNMT were invariable, according to statistical data (Fig. 5B,C). Further, the inhibitor remained within the active site: first, the ligand stays in the active site in the average structure (Fig. 5)—which wouldn’t be expected if the ligand leaves the enzyme—; second, the radius of gyration of complex is smaller than that of the free enzymes, as expected from the ligand being located in an internal cavity of the protein (Fig. 5). To assess energy degeneracy of A and B orientations, protein–ligand interaction was estimated from MD computations. Details of the structure closest to the MD average of CC-410 bound to *m*NNMT are shown in Fig. 5D. MD trajectories of *m*NNMT Auto Dock analysis for the two forms showed that the B and A orientations have only slight differences in energy. Moreover, the same analysis for *m*NNMT-SAH interaction demonstrated that, regardless of orientation, CC-410 binds to NNMT more tightly than to SAH (Table 3). Notably, this happens even when the average number of H-bonds partaken by SAH within the protein is larger. However, the contribution of H-bonds strongly depends on their geometry and the surrounding environment. Figure 5A shows the best docking solutions for CC-410-*h*NNMT and CC-410-*m*NNMT and SAH- *m*NNMT complexes, where SAH fills only the donor pocket of the active site ^25^. By overlapping the aligned structures of CC-410 and SAH (Fig. 5E) we further observed that while the A1 conformation fully overlaps with SAH, acting as a single-substrate inhibitor. However, B2 only overlaps SAH incompletely because CC-410 not only partially occupies the methyl-donor (S-adenosyl-methionine) site of the active site but also fills the acceptor (nicotinamide) pocket, behaving as a bisubstrate-like inhibitor. To illustrate this point, we aligned the A and B orientations of CC-410 with two known NNMT bisubstrate inhibitors MS2756 ^43^ and compound 8 ^40^ (Fig. 5F). Our analysis demonstrated that NNMT-CC-410 binding aligns well with these alternative inhibitory complexes and notably that, depending on its conformation, CC-410 can act as either a single substrate or a bisubstrate mimetic inhibitor. Taken together these data demonstrate that CC-410 is a stable and highly specific inhibitor of NNMT.

**Table 2:** Summary of AutoDOCK Vina results for the interaction between NNMT and CC410.

Target	PDB	Solution	Score	ubRMSD* (Å)	No. H-bonds	Orientation**
*h*NNMT	6b1a	S1	− 3.9	0	2	A1
		S2	− 3.8	8.92	1	B1
		S3	− 3.8	− 3.79	3	A2
*h*NNMT	2iip	S1	− 2.4	0	1	A1
		S2	− 2.2	8.92	1	B1
		S3	− 2.0	8.52	0	B2
*m*NNMT	5yji	S1	− 6.3	0	4	B2
		S2	− 5.1	8.81	0	A1
		S3	23.9	26.85	0	Surface

*Upper-bound RMSD with respect to best solution in each calculation. CC410 has C1 symmetry.

**A1: methyl-pentanoate points to cavity mouth and acetoxy group locates within donor cavity; A2: methyl-pentanoate pointing to cavity mouth and acetoxy group gets inside nicotinamide pocket; B1: acetoxy group pointing to cavity mouth and the methyl-pentanoate group locates within donor cavity; B2: acetoxy group pointing to cavity mouth and methyl-pentanoate group gets inside nicotinamide pocket; Surface: molecule binds out of the active site pocket.

**Table 3:** Summary of Protein–ligand interactions estimated from MD computations.

Target	PDB	Ligand	Orientation	Average no. of ligand H-bonds		∆*E*_VdW_	∆*E*_ELEC_
				As donor	As acceptor	(kcal·mol^−1^)	
*h*NNMT	6b1a	CC-410	A1	2.5 ± 0.9	0.8 ± 0.8	− 30.1 ± 3.8	+ 2.8 ± 12.7
*m*NNMT	5yji	CC-410	B2	1.2 ± 0.8	1.92 ± 0.8	− 30.8 ± 3.4	− 15.0 ± 11.2
	5yji	CC-410	A1	1.8 ± 0.8	0.44 ± 0.6*	− 32.3 ± 3.3	− 1.5 ± 11.0
	5yji	SAH	N.A	4.6 ± 0.9	5.2 ± 1.2	− 16.7 ± 4.5	+ 2.7 ± 14.9

Error values correspond to standard deviations. *All the H-bond data show skewed distributions. No negative values are neither recorded nor expected. N.A. stands for not applicable.

### NNMT is a key regulator of the glucocorticoid-mediated commitment state in the progression from pre-adipocyte to adipocyte

**Figure 6:**
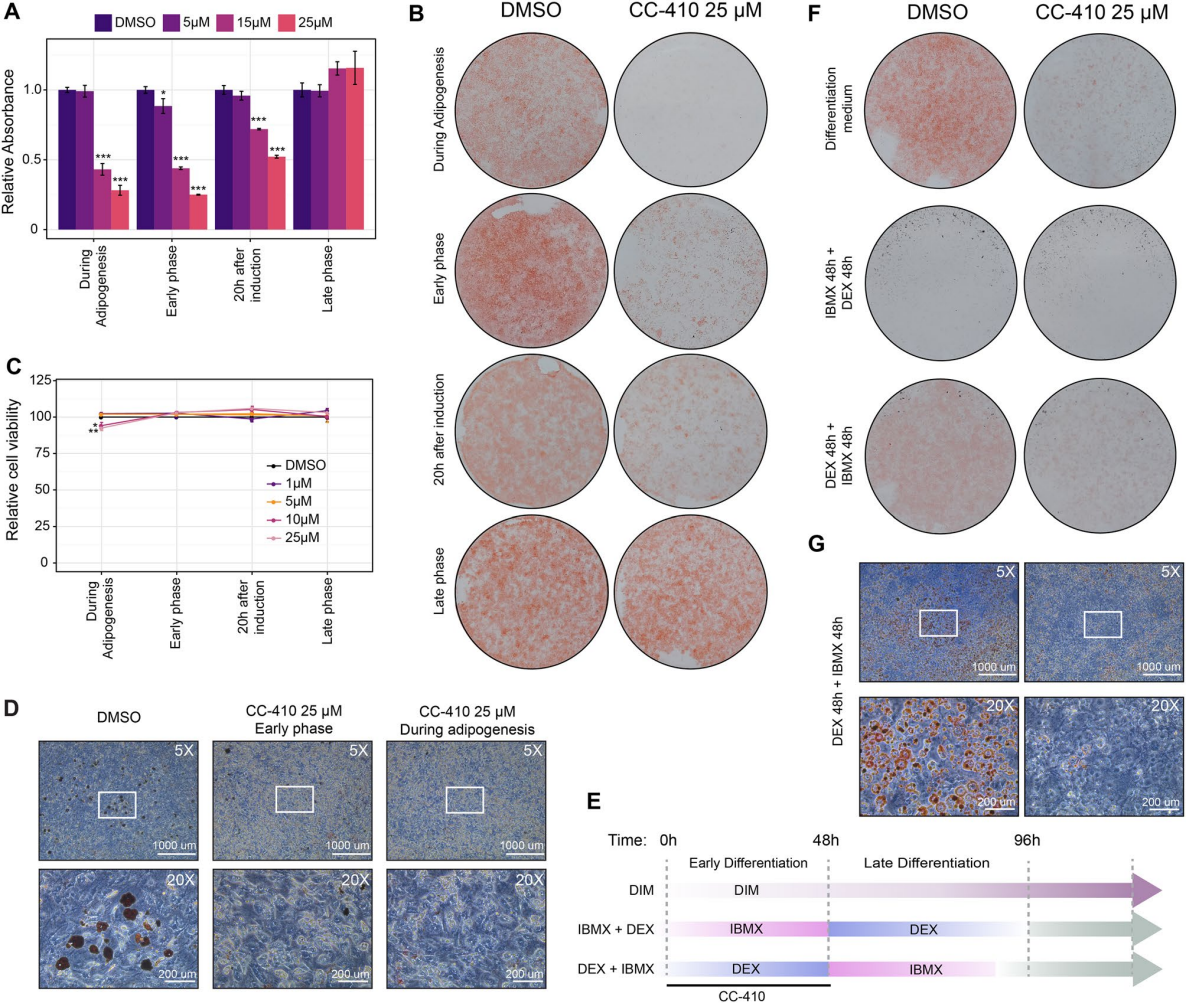
Time-dependent inhibition of NNMT using CC-410 molecule impairs terminal adipocyte differentiation by blocking the GC signaling cascade. (**A**) Post confluent 3T3-L1 cells were incubated with various concentrations of CC-410 with different schedules during the differentiation process. Oil Red-O-staining was assessed 10 days after adipogenic induction and quantified by OD measurement. Data are presented as means ± S.D. (n = 3) versus vehicle control (DMSO). (**B**) Representative images of culture plates following Oil Red-O staining in the untreated controls and CC-410 treated adipocytes. (**C**) Viability of 3T3-L1 cells treated with CC-410 (1–25 uM). Data are presented as average ± SEM normalized (% untreated control) versus CC-410 treated samples. (**D**) CC-410 treatments effectively inhibit adipogenesis in mouse embryonic stem cell precursor cell line C3H10T1/2. Representative microscope images (5× and 20×) of Oil Red-O stained C3H10T1/2 cell lines treated with vehicle control (DMSO) or 25 µM CC-410 in the first 48 h of DIM induction (Early phase) or continued throughout the period of differentiation (During adipogenesis). (**E**) Time schedule administration of the different combinations of hormonal inducers with/without CC-410 in 3T3-L1 cells. (**F**) Two days post-confluent 3T3-L1 were treated with the combinations indicated and stained 10 days later with Oil Red-O-staining. From top to bottom: DIM ± CC-410 48 h, IBMX ± CC-410 48 h followed by 48 h treatment with DEX and DEX ± CC-410 48 h followed by treatment with IBMX for 48 h. (**G**) Microscope images (5× and 20× magnifications) of Oil Red-O-staining of the DEX 48 h + IBMX 48 h condition with and without CC-410 treatment. Data represent three independent experiments each with at least three technical replicates. Statistical significance was calculated using a one-sided Welch’s t-test. For all panels: *p < 0.05; **p < 0.01; ***p < 0.001.

Once we corroborated that CC-410 specifically targets NNMT, the molecule was used to modulate enzyme activity over time. To check if CC-410 was able to inhibit adipogenesis, we firstly determined a range of concentrations where the drug showed the highest anti-adipogenic activity with the lowest cytotoxic effects. We treated post-confluent 3T3-L1 cell line with different drug concentrations throughout the differentiation process (during adipogenesis). Oil Red-O staining quantification showed that concentrations of 15 and 25 µM strongly reduced adipogenesis by 60% and 80% respectively compared to untreated control (Fig. 6A,B). Cell viability assays confirmed staining reduction was not due to the cytotoxic effect of CC-410, indeed at the highest concentration it induced only a slight reduction in cell viability (Fig. 6C). To confirm our previous results, 3T3-L1 pre-adipocytes were incubated with CC-410 at different time points. CC-410 treatment during the early phase (from T0 to 48 h) markedly reduced adipogenesis, with levels analogous to continuous treatment. Although with a slightly lower effect, CC-410 treatment strongly inhibited adipogenesis when administered in the first 20 h after hormonal induction, with 50% inhibition at the highest concentration tested. Importantly, no anti-adipogenic effect was evident when CC-410 was continuously dispensed after the early phase of adipogenesis, when the MCE window is already closed (Fig. 6A,B). This time-sensitive anti-adipogenic effect of CC-410 undeniably confirms that NNMT action is restricted to a discrete time interval from the middle to the end of MCE, concurrent with the cell fate commitment point. Importantly, the anti-adipogenic and phase sensitive effect of CC-410 was further confirmed in the mouse embryonic stem cell precursor cell line C3H10T1/2, demonstrating that the proposed mechanism is cell-type independent (Fig. 6D). It has been demonstrated that DEX provides a signal that primes pre-adipocytes toward differentiation as it activates a signalling cascade that is subsequently necessary for the pro-adipogenic action of IBMX^62^. Consistent with this, we observed that when 3T3-L1 was sequentially treated with DEX for 48 h followed by 48 h treatment with IBMX, the cells underwent terminal differentiation, while the opposite treatment failed to induce differentiation (Fig. 6E,F). Notably, we found that treatment with CC-410 during the 48 h of DEX stimulation strongly impaired adipogenesis, by inhibiting the DEX-primed pre-adipocytes state (Fig. 6F,G), demonstrating that NNMT is a key regulator of the glucocorticoid signalling network early in the commitment phase of adipogenesis.

## Discussion

Disclosing the molecular aspects and timing that coordinate the irreversible lineage commitment point and the molecular players that regulate this well-defined time lag is crucial to prevent hyperplasia and likely the onset and worsening of obesity. Although compelling evidence demonstrated that the genetic and chemical inhibition of NNMT protects against weight gain and associated negative metabolic effects in diet-induced obesity models, the upstream factors regulating its expression and the molecular patterns underling NNMT adipogenic function have not been characterized. By using the standard protocol for 3T3-L1 pre-adipocyte differentiation as an in vitro model that faithfully recapitulates the biology of adipose tissue and whose validity has been extensively corroborated in vivo^4^, we demonstrated that NNMT is an essential early player during the lineage commitment stage.

We demonstrated that CEBPB, one of the initiators of the transcriptional cascade that triggers adipogenesis, transactivated *Nnmt* expression in response to DEX, and that deletion of CEBPB binding sites on *Nnmt* promoter impairs this transactivation. *Nnmt*-KO cell lines did not acquire the characteristic and specialized functions of terminal adipocytes but retained a fibroblast progenitor-like morphology. Accordingly, gene expression analysis demonstrated that depletion of *Nnmt* induces a reduction in the expression of late fat markers like *Pparg*, *Cebpa* and *Lpl* without affecting *Cebpb*.

The link between the decision to terminally differentiate and both cell cycle length, particularly the G1 phase, and properly-timed cell cycle exit has been validated across several stem cell lineages^63–65^. Importantly, it has been demonstrated that cells that enter their final mitosis later do not differentiate^58^. Accordingly, we demonstrated that NNMT controls the adipocyte-commitment stage by regulating the proper timing of the cell cycle phases during MCE. In fact, NNMT deficiency delays permanent withdrawn from the cell cycle and shortens the length of the G1 phase as compared with control cell lines, which do differentiate.

In order to unambiguously elucidate the role of NNMT during adipogenesis we used a complementary pharmacological approach to inhibit NNMT function. Compared to a genetic approach, the chemical inhibition of enzyme activity is an important tool for understanding complex regulatory mechanisms since small molecules allow the modulation of protein function in a tunable- and conditional manner. However, genetic and pharmacological approaches are not always truly equivalent as they may affect a protein’s activity in different ways and will therefore provide non-identical information about the protein’s biological function^66,67^.

In this study we report the chemical and computational characterization of a novel small molecule NNMT inhibitor. We firstly demonstrated that CC-410 stably binds to and highly specifically inhibits NNMT, and then we used it to temporally modulate protein activity during pre-adipocyte differentiation stages. We demonstrated—to our knowledge for the first time—that the chemical inhibition of NNMT at the very early stages of adipogenesis impairs the ability of pre-adipocytes to terminally differentiate, acting by deregulating the glucocorticoid signalling network that primes the adipocyte precursor cells for differentiation. The overlapping results obtained here with both a chemical and a genetic approach, demonstrate the central role of NNMT in this cellular network and may help to define the mechanism of action of NNMTi compounds. In light of these results, NNMT can be considered as a potential therapeutic target not only, as previously described, to treat obesity but also to prevent early-onset and glucocorticoid-induced obesity.

## Data Availability

Raw RNA-Seq fastq files have been deposited at the European Nucleotide Archive (ENA) with the accession number PRJEB55900 (ERP140844); https://www.ebi.ac.uk/ena/browser/view/PRJEB55900.
